# Phase III randomized study of fotemustine and dacarbazine versus dacarbazine with or without interferon-α in advanced malignant melanoma

**DOI:** 10.1186/1479-5876-11-38

**Published:** 2013-02-13

**Authors:** Antonio Daponte, Simona Signoriello, Luigi Maiorino, Bruno Massidda, Ester Simeone, Antonio Maria Grimaldi, Corrado Caracò, Giuseppe Palmieri, Antonio Cossu, Gerardo Botti, Antonella Petrillo, Secondo Lastoria, Ernesta Cavalcanti, Pasquale Aprea, Nicola Mozzillo, Ciro Gallo, Giuseppe Comella, Paolo Antonio Ascierto

**Affiliations:** 1 Department of Melanoma, Istituto Nazionale Tumori Fondazione Pascale, Via Mariano Semmola, 80131, Naples, Italy; 2 Medical Statistics, Second University, Naples, Italy; 3 Medical Oncology, S.Gennaro Hospital Napoli, Naples, Italy; 4 Medical Oncology, University of Cagliari, Cagliari, Italy; 5 Unit of Cancer Genetics, Institute of Biomolecular Chemistry, C.N.R., Sassari, Italy; 6 Department of Pathology, Azienda Ospedaliero Universitaria, Sassari, Italy; 7 Department of Pathology, Istituto Nazionale Tumori Fondazione Pascale, Naples, Italy; 8 Department of Radiology, Istituto Nazionale Tumori Fondazione Pascale, Naples, Italy; 9 Department of Nuclear Medicine, Istituto Nazionale Tumori Fondazione Pascale, Naples, Italy; 10 Laboratory Medicine Unit, Istituto Nazionale Tumori Fondazione Pascale, Naples, Italy; 11 Vascular Access Unit, Istituto Nazionale Tumori Fondazione Pascale, Naples, Italy

**Keywords:** Melanoma, Fotemustine, Dacarbazine, Interferon-α

## Abstract

**Background:**

The effect of the addition of fotemustine and/or interferon (IFN) to standard therapy with dacarbazine alone in patients with advanced malignant melanoma was investigated in a multicenter, randomized 2x2 factorial design trial.

**Methods:**

A total of 260 patients were randomly assigned to one of four treatment groups: (A) fotemustine and dacarbazine repeated on 3-week cycle; (B) same treatment as (A) plus IFN-α2b three times per week; (C) dacarbazine alone repeated on 3-week cycle; (D) same treatment as (C) plus IFN-α2b three times per week. Two comparisons were planned to assess the efficacy of fotemustine (groups A+B vs. C+D) and IFN-α2b (groups A+C vs. B+D).

**Results:**

Addition of fotemustine did not significantly improve overall survival (OS) (p=0.28) or progression-free survival (PFS) (p=0.55); Hazard ratio (HR) for OS was 0.93 (95% CI 0.71-1.21). Similarly, addition of IFN-α2b did not improve OS (p=0.68) or PFS (p=0.65); HR for OS was 0.92 (95% CI 0.70-1.20). Overall response rate was not improved by the addition of either fotemustine (p=0.87) or IFN-α2b (p=0.57). The combination of all three drugs resulted in the highest occurrence of adverse events.

**Conclusions:**

No significant improvement in outcomes were observed with the addition of either fotemustine or IFN-α2b to dacarbazine.

**Trial registration:**

ClinicalTrials.gov: NCT01359956

## Background

The incidence of cutaneous melanoma has risen rapidly over the last 30 years, with an annual rate increase among the Caucasian population of approximately 3%. Melanoma is now the third most prevalent cancer, representing about 7% of tumors in both men and women. The median age at diagnosis for melanoma is 63 years in men and 56 years for women. Although melanoma is rare before the age of 30 years, it is the second and third most commonly diagnosed cancer in women and men, respectively, in the 20 to 29 years age group. The 5-year and 10-year relative survival rates for patients with melanoma are 91.2% and 89.1%, respectively. For those with localized melanoma, the 5-year survival rate is 98.2%; 5-year survival rates for individuals with regional and distant stage disease decline to 61.7% and 15.2%, respectively [1].

Treatment options for patients with advanced melanoma are limited and non-curative in the majority of cases. In a meta-analysis of 42 phase II trials including more than 2100 patients, the median survival time was 6.2 months [95% confidence interval (CI), 5.9−6.5 months), with 25.5% of the patients (95% CI, 23.6−27.4%) alive at 1 year [2]. Median progression-free survival (PFS) was 1.7 months (95%CI, 1.6−1.8 months), with 14.5% of the patients (95% CI, 12.9−16.1%) progression-free at 6 months.

Dacarbazine (DTIC) was the first approved chemotherapeutic agent for the treatment of metastatic melanoma and, for more than 30 years, was the standard treatment for such disease. Fotemustine is the most active nitrosourea in metastatic melanoma, with an objective response rate of 20–25%, with 5–8% of complete responses, and was the first drug to show significant efficacy in brain metastases [3,4]. One hypothesis to explain this is that fotemustine, thanks to the phosphoalanine group, is highly lipophilic and so able to diffuse across the blood–brain barrier. Another explanation could be the potential effect of fotemustine in inhibiting vascular endothelial growth factor (VEGF)-C release and thereby reducing tumor diffusion [5]. In a phase III study, fotemustine was associated with a higher overall response rate (ORR) than dacarbazine in the intention-to-treat (ITT) population (15.2% vs 6.8%, *p*=0.043). However, the response duration (time to disease progression and overall survival [OS]) was not statistically significantly different between groups [6].

Interferon-α (IFN) has been suggested to exert activity against melanoma through immunomodulatory mechanisms [7], although it also has an anti-proliferative effect. Evidence for the involvement of different immunomodulatory mechanisms has been derived from several studies which have shown an increase in tumor infiltrating cells [8], the development of autoantibodies and clinical manifestations of autoimmunity (~30%) [9,10], a decrease in circulating Treg cells [11], modulation of the STAT1/STAT3 balance in tumor cells and host lymphocytes [12], changes in serum cytokine concentrations [13], and normalization of T-cell signaling defects in peripheral blood lymphocytes [14,15]. In the adjuvant setting, a meta-analysis of randomized melanoma trials using a wide range of IFN dose regimens revealed that the benefits of IFN are independent of dose or therapy duration, and translate into an absolute OS benefit of approximately 3% (95% CI: 1–5%) at 5 years [16,17].

In our previous experience [18], 43 patients with advanced melanoma received first-line therapy with a combination of fotemustine 100 mg/m^2^ on day 1, intravenous (IV) dacarbazine 250 mg/m^2^ on days 2–5 every three weeks, and subcutaneous (SC) α2a 3 MIU three times a week until progression. The ORR was 40% (95% CI, 25-56%), and the median duration of response was 24 weeks. Median survival time was 40 weeks, with a 13% 2-year survival rate. Similar results (ORR, 38.3% [95% CI, 26.1–51.8%], median duration of response, 28 weeks; median survival, 36 weeks) were observed in a subsequent study in which cisplatin was added to fotemustine, dacarbazine and IFN [19], suggesting the addition of cisplatin was not clinically beneficial. In both these studies, some patients achieved a durable complete response (CR). On this basis, and supported by findings from other studies [20], we considered that IFN may have contributed to prolonging the median duration of response (by about 6 months in both studies).

In this prospective, randomized, controlled study, we assessed the effect of adding fotemustine and/or IFN to standard therapy with dacarbazine alone in patients with advanced malignant melanoma.

## Methods

### Patients and procedures

This multicenter, randomized, open-label, 2×2 factorial phase III trial (ClinicalTrials.gov NCT01359956) compared fotemustine and dacarbazine versus dacarbazine with or without the addition of α in patients with advanced malignant melanoma. The study was designed, co-ordinated and conducted independently by the investigators and was approved by the Ethical Committees of participating institutions.

Adult (>18 years old) patients with malignant melanoma (histologically confirmed) in advanced stage or recurrent after surgery, ECOG performance status (PS) 0–2 and not amenable to further surgery or local therapy were eligible if they had adequate bone marrow function, normal liver and renal function, a life expectancy greater than 3 months and no prior surgery in the previous 3 weeks. Only untreated patients were eligible. Palliative radiation, if required, could be performed before starting chemotherapy. If palliative radiation was required during the study, the patient was permanently discontinued from further treatment. Female patients were eligible if they were not pregnant or lactating. Patients with known HIV disease, other previous or concurrent malignancies (except for surgically cured carcinoma *in-situ* of the cervix and basal or squamous cell carcinoma of the skin), prior chemo-immunotherapy, concurrent treatment with other experimental drugs, chemotherapy, immunotherapy, hormone therapy and radiation therapy were excluded. All patients provided written informed consent.

Patients were randomly assigned to one of four treatment groups: (A) fotemustine 100 mg/m^2^ IV on day 1 and dacarbazine 900 mg/m^2^ IV on day 2 repeated on a three-week cycle; (B) same treatment as (A) plus α2b 5 MUI three times per week; (C) dacarbazine alone 900 mg/m^2^ IV on day 1 repeated on a three-week cycle; (D) same treatment as (C) plus α2b 5 MUI three times per week. Patients were randomized through a computerized procedure of permuted blocks centralized at the coordinating center (Medical Oncology, NCI Napoli), stratified by PS (0–1,2) and site of metastases (visceral, not visceral).

Fotemustine was administered in a 250 ml DW5 (protected from light) as a 1-hour infusion and dacarbazine was delivered in 500 ml of saline solution by a 1-hour infusion. Antiemetic prophylaxis with 5-HT3 receptor antagonists was routinely used. Courses were repeated every three weeks. After three cycles, a rest period of five weeks was required to ensure complete hematological recovery. Disease assessment (measurement of all tumor lesions) was performed by CT scan before receiving therapy, every three cycles, at the end of treatment and every three months during the follow-up period.

### Statistical analysis

To identify a hazard ratio (HR) of 0.65 for each of the two comparisons, with a two-tailed type I error α=5% and a type II error β=10% (power = 90%), 227 deaths were needed and 270 patients were planned to be enrolled. These assumptions were based on two our previous phase II trials [18,19].

Two comparisons were planned, combining the treatment groups in a 2x2 manner: (i) to assess the efficacy of fotemustine, groups A + B were compared with groups C + D; and (ii) to assess the efficacy of IFN, groups A + C were compared with groups B + D. Accordingly, except for baseline values, results are reported separately for the two comparisons.

The primary endpoint was OS, defined as the time from the date of randomisation to the date of death from any cause, or the date of last follow-up for living patients. Secondary end points were PFS, response, and toxicity. PFS was defined as the time from the date of randomisation to the date of progression of disease or death from any cause, whichever occurred first, or date of last follow-up for patients without progression and alive at the end of the study. Best response was defined according to the WHO criteria [21] and was assessed every three courses during treatment. CR was defined as disappearance of all symptoms and signs of all measurable disease, lasting for at least four weeks, without appearance of new lesions. Partial response (PR) was defined as a >50% reduction in the sum of the products of the perpendicular diameters of all measurable lesions, lasting for at least four weeks, without appearance of new lesions or enlargement of existing lesions. Progressive disease (PD) was defined as an increase in the product of two perpendicular diameters of any measured lesion by >25% over the size present at entry on study, or the appearance of new lesions. All other patients were considered to have stable disease (SD). Overall response rate (ORR) included CR and PR. Toxicity was scored according to the WHO classification [21], with events recorded as per the worst severity for each patient.

All efficacy analyses were done on an ITT basis. Significance level was 0.05 (two-tailed) without adjustment for multiple comparisons. OS and PFS curves were estimated with the Kaplan-Meier (K-M) method, and treatments were compared with a two-sided log-rank test. HR and 95% CIs were estimated by a Cox proportional hazards model that included treatment, gender, PS, site of metastases, Breslow score and the other treatment factor as covariates. Further exploratory analyses of OS were planned for predefined subgroups of patients.

Contingency tables and *χ*^2^ test were applied in the assessment of response. All patients who received at least one dose of treatment were included in the statistical analysis of toxicity: first, an exact linear permutation test was applied to compare all toxicity grades, second, an exact *χ*^2^ test was applied that compared severe (grades 3–5) versus not severe (grades 0–2) toxicity.

## Results

### Patients

**Figure 1:**
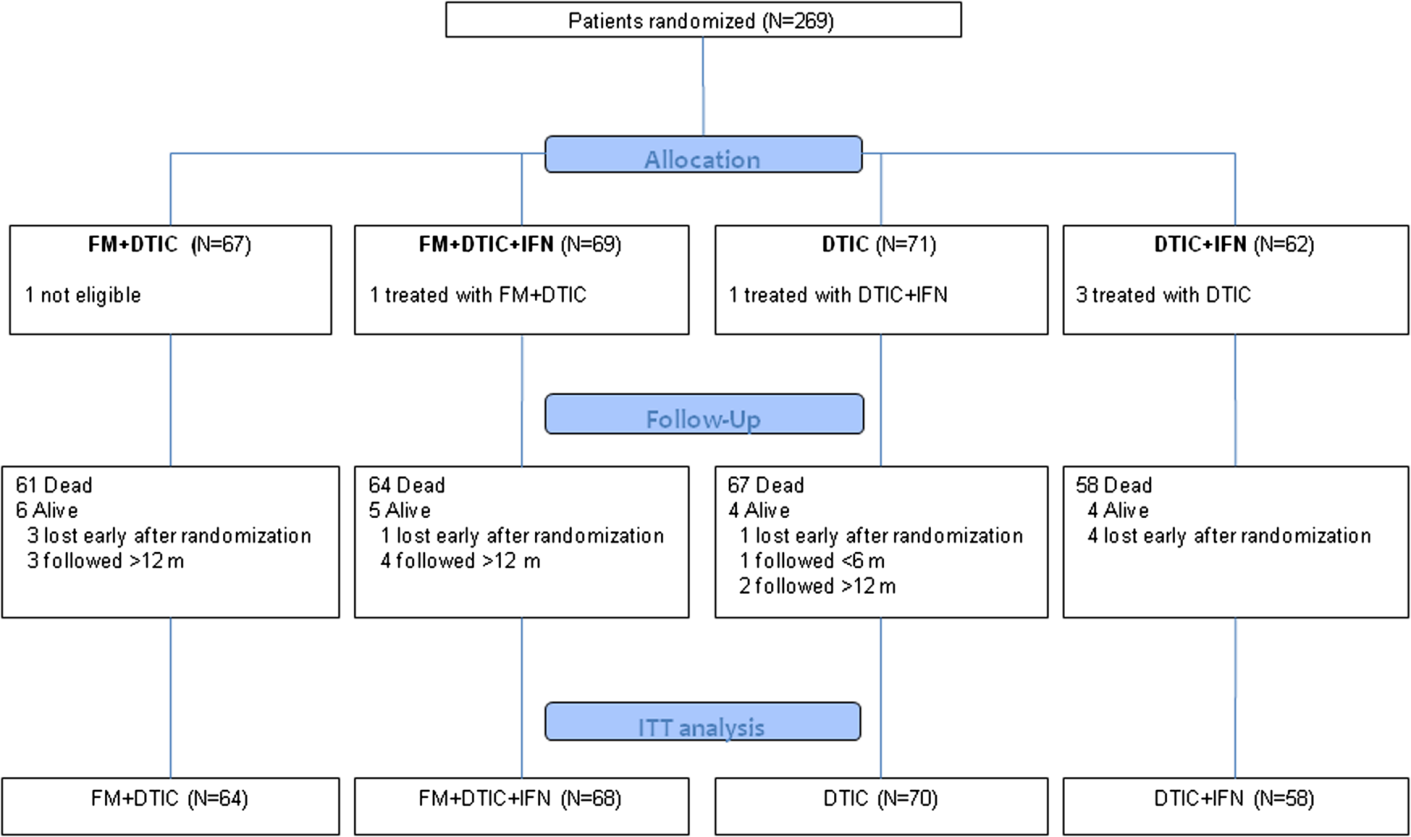
Study flow diagram according to CONSORT.

A total of 269 patients with advanced malignant melanoma were randomized between March 2002 and July 2007. Nine patients were lost to follow-up immediately after randomization. Five patients received different treatment from that assigned. One patient was found to be ineligible after randomization. All the patients, with the exclusion of those lost after randomization for which no information was available, were analyzed according to the ITT approach. Participant flow is described in Figure 1.

Baseline characteristics of the 260 patients were generally similar across the four treatment arms, although minor differences were observed in adjuvant immunotherapy, LDH and presence of brain metastases (Table 1). Median age of patients was 55 years (mean ± SD, 55 ± 15 years). Almost all patients had a PS of 0–1 and almost half had a Breslow score between 2 and 4. Most patients were classified as M1C with regard to metastatic site.

**Table 1:** Baseline characteristics of patients by treatment arm

**Variable**	**FM+DTIC**	**FM+DTIC+IFN**	**DTIC**	**DTIC+IFN**	**Total**
	**(*N*=64)**	**(*N*=68)**	**(*N*=70)**	**(*N*=58)**	**(*N*=260)**
**Male gender**	42 (66%)	35 (51%)	42 (60%)	38 (66%)	157 (60%)
**Age***years*, mean (SD)	54 (13)	50 (15)	59 (15)	56 (14)	55 (15)
**Adjuvant immunotherapy**					
Yes	20 (31%)	24 (35%)	21 (30%)	8 (14%)	73 (28%)
No	43 (67%)	43 (63%)	49 (70%)	49 (84%)	184 (71%)
Missing	1 (2%)	1 (1%)	0 (0%)	1 (2%)	3 (1%)
**Performance Status**					
0	45 (70%)	52 (76%)	55 (79%)	41 (71%)	193 (74%)
1	16 (25%)	12 (18%)	14 (20%)	15 (26%)	57 (22%)
2	2 (3%)	4 (6%)	1 (1%)	1 (2%)	8 (3%)
Missing	1 (2%)	0 (0%)	0 (0%)	1 (2%)	2 (1%)
**Histology**					
Superficial spreading melanoma	13 (20%)	8 (12%)	7 (10%)	6 (10%)	34 (13%)
Nodular melanoma	27 (42%)	36 (53%)	36 (51%)	31 (53%)	130 (50%)
Lentigo malignant melanoma	0 (0%)	0 (0%)	1 (1%)	0 (0%)	1 (0%)
Acral lentiginous melanoma	1 (2%)	2 (3%)	5 (7%)	1 (2%)	9 (3%)
Other	22 (34%)	22 (32%)	21 (30%)	19 (33%)	84 (32%)
Missing	1 (2%)	0 (0%)	0 (0%)	1 (2%)	2 (1%)
**Breslow**					
<2	18 (28%)	17 (25%)	10 (14%)	10 (17%)	55 (21%)
≥2 and ≤4	30 (47%)	27 (40%)	36 (51%)	28 (48%)	121 (47%)
>4	15 (23%)	24 (35%)	24 (34%)	20 (34%)	83 (32%)
Unknown	1 (2%)	0 (0%)	0 (0%)	0 (0%)	1 (0%)
**Ulceration**					
Yes	28 (44%)	27 (40%)	28 (40%)	24 (41%)	107 (41%)
No	23 (36%)	29 (43%)	35 (50%)	27 (47%)	114 (44%)
Unknown	13 (20%)	12 (18%)	7 (10%)	7 (12%)	39 (15%)
**LDH**					
Normal	28 (44%)	31 (46%)	23 (33%)	17 (29%)	99 (38%)
>1 UNL and ≤ 2UNL	25 (39%)	24 (35%)	19 (27%)	22 (38%)	90 (35%)
>2 UNL	11 (17%)	13 (19%)	28 (40%)	19 (33%)	71 (27%)
**Visceral disease**					
Yes	51 (80%)	49 (72%)	53 (76%)	47 (81%)	200 (77%)
No	12 (19%)	19 (30%)	17 (24%)	10 (17%)	58 (22%)
Missing	1 (2%)	0 (0%)	0 (0%)	1 (2%)	2 (1%)
**Site of metastases**					
M1A	6 (9%)	11 (16%)	6 (9%)	3 (5%)	26 (10%)
M1B	8 (12%)	2 (3%)	7 (10%)	4 (7%)	21 (8%)
M1C	49 (77%)	55 (81%)	57 (81%)	50 (86%)	211 (81%)
Missing	1 (2%)	0 (0%)	0 (0%)	1 (2%)	2 (1%)
**Brain metastases**					
Yes	11 (17%)	13 (19%)	4 (6%)	5 (9%)	33 (13%)
No	53 (83%)	55 (81%)	66 (94%)	53 (91%)	227 (87%)

Data are reported as absolute numbers (%), except for age (mean and standard deviation).

FM = fotemustine; DTIC = dacarbazine; IFN = interferon-α2b.

154 out the 260 patients discontinued the treatment in the first three cycles, mostly because of progression (74%) or death (17%). Only in five case (3%) treatment was discontinued because of toxicity or refusal, mainly in the three-drug arm.

### Efficacy analysis

**Figure 2:**
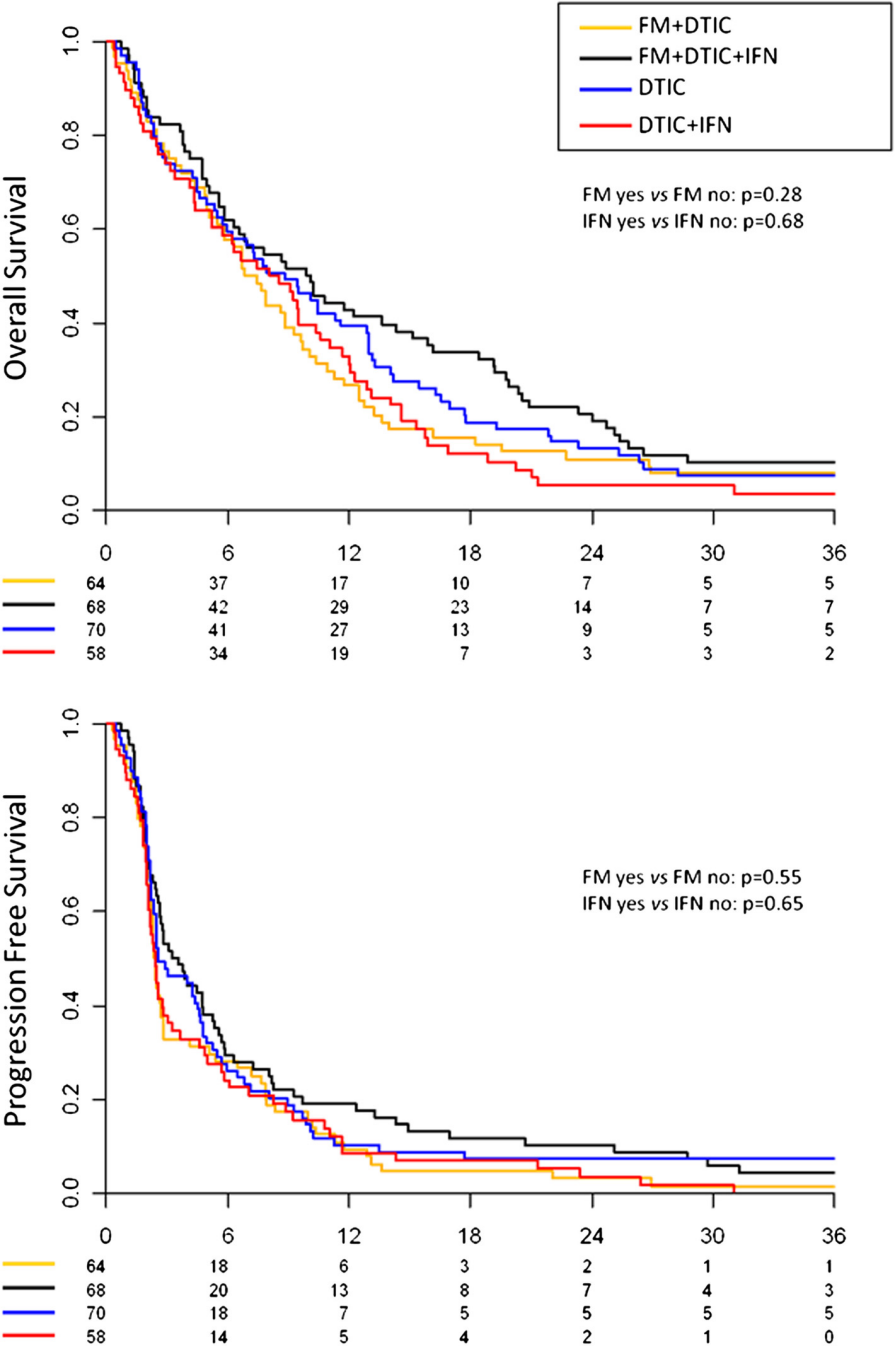
Kaplan-Meier curves of the four treatment arms: overall survival (upper panel) and progression-free survival (lower panel).

All 260 patients were included in the efficacy analyses. Overall, 250 deaths and 253 disease progressions were observed. K-M curves of the four treatment arms are shown in Figure 2 for OS (upper panel) and PFS (lower panel). Results of the efficacy analysis are reported in Table 2.

**Table 2:** Efficacy outcomes

	**Fotemustine efficacy**			**Interferon-α2b efficacy**		
	**FM/DTIC/IFN + FM/DTIC**	**DTIC/IFN + DTIC**	HR* (95% CI)	**FM/DTIC/IFN + DTIC/IFN**	**FM/DTIC + DTIC**	HR* (95% CI)
**Overall survival:**						
Events, n (%)	125 (95%)	125 (98%)		122 (97%)	128 (96%)	
Median, months (95% CI)	7.9 (6.6−10.2)	8.6 (6.3−10.4)	0.93 (0.71−1.21)	9.1 (6.3−11.1)	7.7 (6.3−9.7)	0.92 (0.70−1.20)
**Progression-free survival:**						
Events, n (%)	128 (97%)	125 (98%)		123 (98%)	130 (97%)	
Median, months (95% CI)	2.7 (2.4−3.8)	2.5 (2.3−3.7)	0.93 (0.72−1.21)	2.8 (2.4−3.9)	2.5 (2.3−2.9)	0.96 (0.73−1.25)
**Overall response, n (%)**	32 (34%)	33 (26%)		34 (27%)	31 (23%)	
(95% CI)	(17−32%)	(18−33%)		(19−35%)	(16−30%)	

* After adjustment by age, sex, performance status, site of metastases, Breslow score and other treatment factor.

FM = fotemustine; DTIC = dacarbazine; IFN = interferon-α2b.

#### Fotemustine

Median OS was 7.9 months (95% CI 6.6−10.2) for patients receiving fotemustine (groups A + B) compared with 8.6 months (95% CI 6.3−10.4) without fotemustine (groups C + D) (*p*=0.28). Median PFS was 2.7 months (95% CI 2.4−3.8) with fotemustine and 2.5 months (95% CI 2.3−3.7) (*p*=0.55) without. In multivariate analysis, adjusted for age, sex, PS, site of metastases, Breslow score and IFN treatment, HR of death and disease progression for patients treated with fotemustine were 0.93 (95% CI 0.71−1.21) and 0.93 (95% CI 0.72−1.21), respectively. No significant difference was seen in ORR between patients who were treated with fotemustine and those who were not (24% [95% CI 17−32%] versus 26% [95% CI 18−33%], *p*=0.87).

#### IFN-α2b

Median OS was 9.1 months (95% CI 6.3−11.1) with α2b (groups B + D) and 7.7 months (95% CI 6.3−9.7) without (groups A + C) (*p*=0.68). Median PFS was 2.8 months (95% CI 2.4−3.9) in α2b-treated patients compared with 2.5 months (95% CI 2.3−2.9) without α2b (*p*=0.28). In multivariate analysis, HR of death and progression for patients treated with α2b were 0.92 (95% CI 0.70−1.20) and 0.96 (95% CI 0.73−1.25), respectively. ORR was 27% (95% CI 19−35%) in patients who received α2b compared with 23% (95% CI 16−30%) in patients who did not (*p*=0.57).

**Figure 3:**
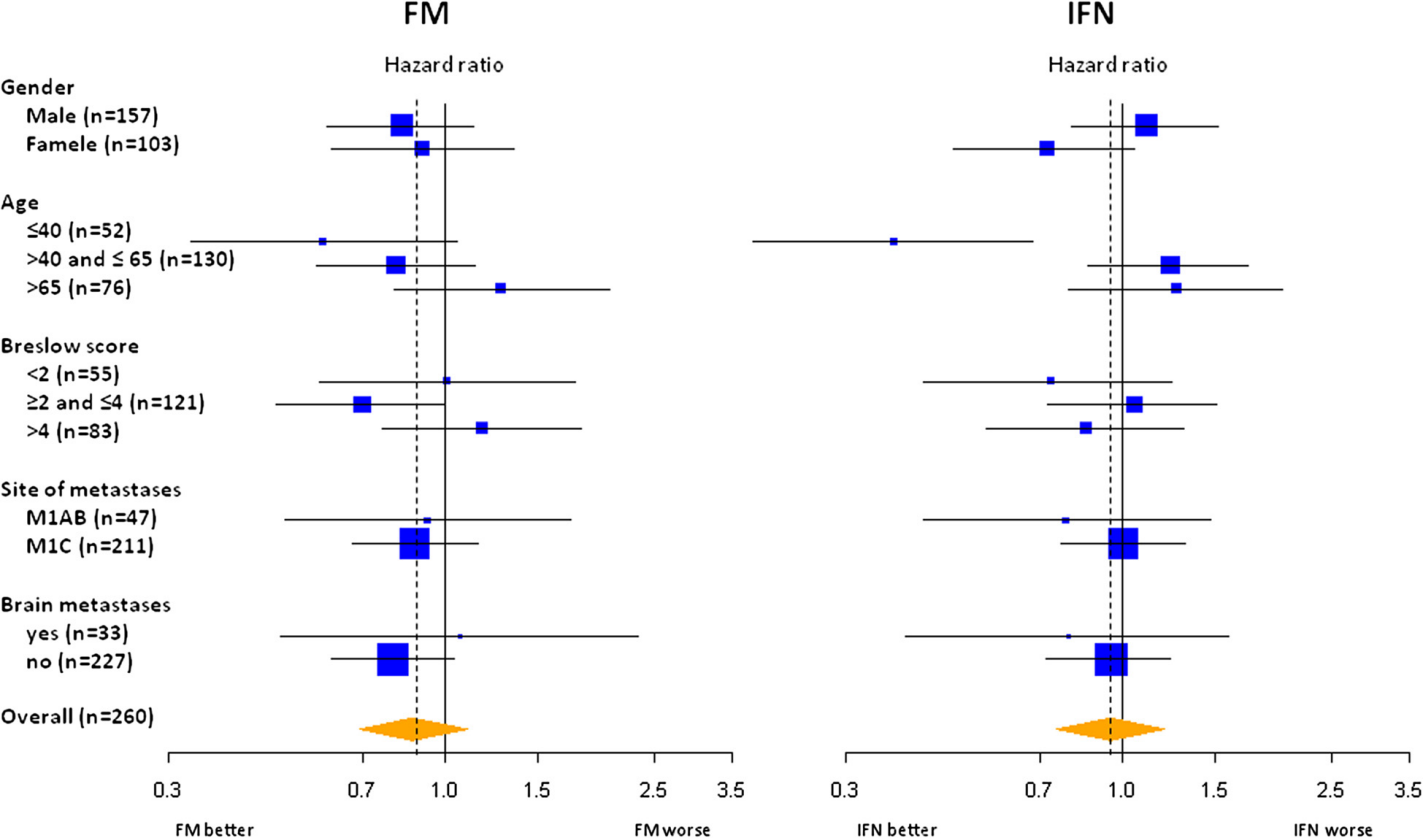
**Overal survival univariate analyses of the effect of Fotemustine (FM, left panel) and Interferon-α2b (IFN, right panel) within subgroups of patients. **The area of each square is proportional to the size of the subgroup; horizontal lines depict 95% confidence intervals of the hazard ratio estimates.

Results of univariate analyses of OS in predefined subgroups of subjects for the two comparisons are reported in Figure 3. No evidence of heterogeneity was suggested by these results, except for age, where increased efficacy in younger subjects was observed both for fotemustine (*p*=0.015 at interaction test) and α2b (*p*<0.001 at interaction test).

Adverse events are reported in Table 3. Overall, a greater number of toxic effects were reported in patients who received all three drugs (Group B). Patients given fotemustine had a significantly higher incidence of anemia and platelet reduction, although the worst degree was generally not severe. Patients given α2b had a significantly higher incidence of neutropenia, anemia and fever. However, similarly to fotemustine, the worst reported degree was not usually severe.

**Table 3:** Worst degree of toxic events

	**Treatment**																				**FM(yes/no)**		**IFN (yes/no)**	
	**FM+DTIC (N=62), n(%)**					**FM+DTIC+IFN (N=67), n(%)**					**DTIC (N=72), n(%)**					**DTIC+IFN (N=52), n(%)**					**p-value**			
	**0**	**1**	**2**	**3**	**4**	**0**	**1**	**2**	**3**	**4**	**0**	**1**	**2**	**3**	**4**	**0**	**1**	**2**	**3**	**4**	**WMW**	**Fisher**	**WMW**	**Fisher**
***Neutropenia***	50(81)	-	7(11)	1(2)	1(2)	37(55)	8(12)	9(13)	7(10)	3(5)	61(85)	1(1)	2(3)	3(4)	2(3)	36(69)	4(8)	4(8)	2(4)	-	0.013	0.421	<0.001	0.192
***Febrile/Infection Neutropenia***	58(94)	1(2)	-	-	-	62(93)	1(2)	-	-	1(2)	69(96)	-	-	-	-	46(89)	-	-	-	-	0.093	0.973	0.476	0.940
***Platelets***	45(73)	4(7)	7(11)	2(3)	1(2)	41(61)	7(10)	9(13)	2(3)	5(8)	66(92)	-	2(3)	1(1)	-	42(81)	3(6)	1(2)	-	-	<0.001	0.018	0.030	0.381
***Anaemia***	55(89)	2(3)	1(2)	1(2)	-	51(76)	5(8)	5(8)	2(3)	1(2)	68(94)	1(1)	-	-	-	44(85)	2(4)	-	-	-	0.002	0.148	0.007	0.510
***Nausea/Vomiting***	47(76)	9(15)	2(3)	1(2)	-	39(58)	22(33)	2(3)	-	1(2)	53(74)	10(14)	6(8)	-	-	33(64)	7(14)	4(8)	2(4)	-	0.604	0.662	0.039	0.510
***Diarrhoea***	56(90)	3(5)	-	-	-	60(90)	4(6)	1(2)	-	-	66(92)	3(4)	-	-	-	46(89)	-	-	-	-	0.156	0.604	0.960	0.243
***Stomatitis***	57(92)	2(3)	-	-	-	63(94)	2(3)	-	-	-	67(93)	1(1)	1(1)	-	-	45(87)	1(2)	-	-	-	0.790	0.604	0.842	0.243
***Neurological***	57(92)	2(3)	-	-	-	65(97)	-	-	-	-	69(96)	-	-	-	-	46(89)	-	-	-	-	0.174	0.604	0.189	0.243
***Hair loss***	57(92)	1(2)	-	1(2)	-	62(93)	2(3)	1(2)	-	-	68(94)	-	1(1)	-	-	43(83)	1(2)	-	2(4)	-	0.843	0.953	0.219	0.894
***Heart general***	59(95)	-	-	-	-	65(97)	-	-	-	-	69(96)	-	-	-	-	46(89)	-	-	-	-	-	0.604	-	0.243
***Liver***	57(92)	1(2)	-	1(2)	-	61(91)	-	-	2(3)	2(3)	67(93)	-	1(1)	1(1)	-	45(87)	1(2)	-	-	-	0.353	0.247	0.562	0.547
***Kidney***	59(95)	-	-	-	-	65(97)	-	-	-	-	67(93)	-	1(1)	-	-	46(89)	-	-	-	-	0.301	0.604	0.354	0.243
***Pulmonary***	59(95)	-	-	-	-	64(96)	1(2)	-	-	-	69(96)	-	-	-	-	46(89)	-	-	-	-	0.340	0.604	0.287	0.243
***Allergy***	59(95)	-	-	-	-	65(97)	-	-	-	-	69(96)	-	-	-	-	46(89)	-	-	-	-	-	0.604	-	0.243
***Fever***	58(94)	-	1(2)	-	-	47(70)	7(10)	10(15)	1(2)	-	66(92)	1(1)	1(1)	1(1)	-	34(65)	9(17)	3(6)	-	-	0.535	0.508	<0.001	0.546
***Other***	58(94)	-	1(2)	-	-	65(97)	-	-	-	-	68(94)	-	-	1(1)	-	43(83)	1(2)	2(4)	-	-	0.141	0.604	0.131	0.243

## Discussion

In this randomized trial we did not find any significant improvement in PFS, OS or ORR with the addition of either fotemustine or IFN-α2b to dacarbazine. The absence of any possible synergism between dacarbazine and fotemustine in our study is consistent with other studies of combination chemotherapy in advanced melanoma. Moreover, there does not appear to be any potential delaying effect on the occurrence of brain metastases through the addition of fotemustine, as was reported by Avril et al. [6]. Moreover, despite our preliminary results which suggested a potential benefit of IFN [18,19], its addition did not improve outcomes in this study. Therefore, the combination of IFN, as immunomodulating and/or anti-proliferative agent, and chemotherapy in the hope of producing additive or synergistic effects was disappointing and the single-agent chemotherapy with dacarbazine was found the treatment with the highest response rate in our series. These findings are consistent with previous studies in which different combination chemotherapy schedules have failed to show superiority in terms of OS compared with dacarbazine alone. In a systematic review of 41 randomised studies in disseminated malignant melanoma, the only advantage over dacarbazine observed with some combination chemotherapy schedules was an increase in response rate [22].

Regarding our study, It should be noted that fotemustine was given every three weeks, rather than being administered at the usual schedule that includes an induction phase (weekly for three weeks) followed by a maintenance phase (same dosage every 3 weeks, 4–6 weeks after the induction phase). Moreover, we also enrolled patients with brain metastases usually excluded from other trials on metastatic melanoma.

This trial was designed and initiated in 2002, before clinical breakthroughs represented by targeted therapies and immunotherapeutic approaches have revolutionized the treatment of advanced melanoma. Indeed, both ipilimumab (a fully human monoclonal antibody that blocks CTLA-4 to promote antitumor immunity) and vemurafenib (a potent inhibitor of mutated V600E BRAF) have been recently approved in Europe and the US for the treatment of metastatic melanoma. Compared with dacarbazine, ipilimumab has been shown to improve OS in a randomized trial in patients with previously treated metastatic melanoma [23], while vemurafenib improved OS and PFS in a randomized trial in patients with previously untreated melanoma harboring the V600 BRAF mutation [24]. Further positive results with ipilimumab were shown in another randomized trial, in which OS was significantly longer in treatment-naïve patients with advanced melanoma treated with dacarbazine plus ipilimumab compared with dacarbazine plus placebo (11.2 months vs 9.1 months) [25].

In addition, ipilimumab has also been assessed in combination with fotemustine in the NIBIT-M1 trial [26], a phase II open-label, single-arm study involving patients with unresectable stage III or IV cutaneous melanoma. The main endpoint of the study was to assess the immune-response disease control rate (irDCR: CR, PR or SD) using immune-related tumor response criteria [27]. A total of 86 patients, 20 of whom had brain metastases, were enrolled in this trial. IrDCR was 46.5% (95% CI, 35.7−57.6%), 1-year OS rate was 52.6% (95% CI, 41.8−63.4), and median OS was 13.3 months (95% CI, 8.9−19.9). These preliminary results suggest that the combination of ipilimumab plus fotemustine is effective and safe in patients with melanoma, although this needs to be confirmed in a randomized trial.

Considering the promise of combination therapy with dacarbazine plus ipilimumab [25] and fotemustine plus ipilimumab [26], the addition of ipilimumab to the combination of fotemustine with dacarbazine could theroretically represent a potential therapeutic option that could benefit from any synergistic effect of the combination chemotherapy (dacarbazine plus fotemustine) as evidenced in our previous phase II studies [18,19]. Unfortunately, our results in the phase III study do not support this hypothesis. The combination of chemotherapy with the BRAF inhibitors (vemurafenib, dabrafenib) still needs to be investigated.

Recent advances in treatments have suggested new strategies for designing rational therapeutic combinations for metastatic melanoma. The combination of vemurafenib and ipilimumab is currently being tested in phase I, as the combination of ipilimumab and anti-PD1, while the combination of BRAF and MEK inhibitors is the most promising treatment for the BRAF-mutated population. In fact, data recently presented [28] showed a PFS of 10.8 months in V600 BRAF-mutant patients receiving dabrafenib plus trametinib. This combination is now being tested in a phase III trial versus vemurafenib in BRAF-mutated patients with metastatic melanoma (NCT01400451).

The treatment of melanoma has the potential to become a model for cancer therapy. An approach similar to HIV treatment, using drugs with different mechanisms of action, for example acting on the MAPK pathway, PI3K-mTOR pathway, apoptosis pathway and immunological monoclonal antibodies, could be considered. A combination approach could consider novel agents together with standard chemotherapy agents. Sequential administration of different agents may inhibit cancer cell growth at different check points, while other agents may inhibit neo-angiogenesis, survival of malignant cells or metastatization [29].

## Conclusions

The addition of either fotemustine or α2b to dacarbazine failed to produce any improvement in outcomes in this study. Considering the promising results of recent trials with targeted therapies and/or immunomodulating antibodies in metastatic melanoma, it seems anachronistic and unwarranted to further investigate combination chemotherapy compared with single agent chemotherapy in these patients.

## Competing interests

PAA is consultant for Merck Sharp & Dohme and Bristol-Myers Squibb. He has participated in advisory boards for Bristol-Myers Squibb, Merck Sharp & Dohme, Roche-Genentech, GlaxoSmithKline, Amgen, Celgene, Medimmune, and Novartis and has received honoraria from Bristol-Myers Squibb, Merck Sharp & Dohme, and Roche-Genentech. ES has received honoraria from Bristol-Myers Squibb. All other authors have no competing interest.

## Authors’ contributions

AD, SS, GP, GC, and PAA, designed the study. AD, LM, BM, ES, AMG, CC, GP, AC, GB, AP, SL, EC, PA, NM, GC, and PAA collected data. SS and CG performed statistical analyses. AD, SS, CG and PAA drafted the manuscript. All authors approved the final manuscript.
